# Multiyear changes in snowmelt phosphorus runoff with soil P drawdown or application of struvite to an organic forage crop

**DOI:** 10.1002/jeq2.70104

**Published:** 2025-10-23

**Authors:** Henry Wilson, Kokulan Vivekananthan, Merrin Macrae, Jane Elliott, Kim Schneider, Joanne Thiessen Martens, Aaron Glenn

**Affiliations:** ^1^ Agriculture and Agri‐Food Canada, Science and Technology Branch, Brandon Research and Development Centre Brandon Manitoba Canada; ^2^ Department of Plant Agriculture University of Guelph Guelph Ontario Canada; ^3^ Department of Geography and Environmental Management University of Waterloo Waterloo Ontario Canada; ^4^ Environment and Climate Change Canada, National Hydrology Research Centre Saskatoon Saskatchewan Canada; ^5^ Department of Soil Science University of Manitoba Winnipeg Manitoba Canada

## Abstract

In this study, changes in P runoff losses from three field‐scale small watersheds in the Northern Great Plains region (Manitoba, Canada) under organic management were evaluated over a 7‐year period (2017–2023). A perennial forage crop was grown through this time period, either with biennial addition of a slow‐release fertilizer to maintain soil fertility (struvite, 4 site‐years) or without P amendment (drawdown, 8 site‐years). Annually measured soil P (Olsen P) strongly correlated with mass balance of P added as struvite or removed by crops and surface runoff. As a result, P limitation of forage yield was observed in the later years of the drawdown treatment. P loads and flow‐weighted mean concentrations (FWMCs) measured in snowmelt runoff after struvite application were compared to predicted values using published regional models developed with 82 site‐years of data from field‐scale small watersheds, with Olsen P, water yield, and percent surface cover as predictors. To evaluate response to struvite over a wider range of conditions, P runoff was measured in two additional watersheds under conventional annual grain production following struvite application (2 site‐years). Higher risk of P loss was observed with higher Olsen P. However, following struvite applications, the risk of P loss to snowmelt was consistently lower than predicted (between −0.13 and −0.85 mg L^−1^ for FWMC of total dissolved P) suggesting that the solubility of struvite in runoff water may be lower than for other forms of soil P also extractable as Olsen P.

## Core Ideas

- Drawdown of surface soil P with repeated harvest of perennial hay reduces potential for snowmelt runoff losses.
- Struvite added to soils significantly increased 0–5 cm Olsen P (147 ± 85%).
- Risk of P loss with snowmelt runoff relates more strongly to background Olsen P than to the level after struvite amendment.
- Further research is needed to determine predictors of change in solubility of added struvite P over time.
- At the same level of Olsen P, risk of P loss with snowmelt is lower than predicted if soils contain struvite.

## Plain Language Summary

Too much phosphorus (P) in lakes and rivers causes algal blooms. Too little P in soils can limit the ability to grow crops. Struvite is a type of P that can be recovered as fertilizer from wastewater. This research evaluates two approaches to managing P losses with snowmelt: (1) growing perennial hay crops to reduce soil P, and (2) using struvite to maintain soil fertility in a low P input cropping system. P in runoff decreased over time with harvests of perennial hay crops. P in runoff after struvite application was lower than expected when compared to fields with similar fertility without struvite, but P in excess of crop needs remained in the soil after harvest. Both strategies offer benefits for water quality with struvite providing the added benefit of recovering P from wastewater. Careful balancing of P inputs with crop needs is required to maintain productivity but prevent accumulation of excess soil P.

**Abbreviations**
DRP: dissolved reactive phosphorus
FWMC: flow weighted mean concentration
MAP: monoammonium phosphate
TDP: total dissolved phosphorus
TP: total phosphorus

## 1 INTRODUCTION

The drawdown of soil P levels has the potential to reduce soil P in fields with elevated concentration and reduce losses of P in runoff (Liu, Elliott, et al., 2019; Withers et al., 2020). However, implementing drawdown strategies requires understanding of trade‐offs for crop production and avoiding the depletion of plant‐available P to concentrations that compromise crop yield. Given equipment limitations and importance of available early‐season plant growth, P fertilizer is rarely applied below the 5‐cm depth. As a result, if higher rates of fertilizer application are utilized to ensure within‐season crop P availability or to rebuild very low background soil P, stratification can occur with a disproportionate accumulation of P in the top 5 cm of the soil profile (H. Wilson et al., 2019, 2025).

The use of less soluble sources of P to maintain or build soil P is a management practice with the potential to reduce accumulation of mobile P near the soil surface and consequently reduce subsequent losses with runoff. Struvite (NH_4_MgPO_4_·6H_2_O) recovered from human or livestock waste streams is a P source receiving increasing attention for use in both conventional and organic field crop production as a low solubility but bioavailable slow‐release fertilizer (Leon et al., 2024; Talboys et al., 2016; Thiessen Martens et al., 2022). Recent research into mobility of P with leaching from soils receiving either granular struvite or a more widely used soluble P source (monoammonium phosphate [MAP]) in Illinois and Ontario has confirmed that application of struvite has the potential to reduce leaching losses while still supplying field crops with adequate fertility (Kokulan et al., 2024; Leon et al., 2024). In addition, low rates of struvite dissolution have been observed in soils under cold conditions during the non‐growing season (Gu et al., 2021; Thiessen Martens et al., 2023), which may reduce the potential for losses in the early growing season (Kokulan et al., 2024) or with fall applications (Leon et al., 2024). Despite a growing body of research with focus on agronomy, soil chemistry, and potential for leaching with the use of struvite as a soil P amendment, there has not yet been an evaluation of the impact of struvite application on P losses with surface runoff (either in rainfall or snowmelt) at the field scale. In addition, very few multiyear studies of the impact of struvite application on agronomy, soils, or water quality have been completed, but there is some evidence that residual effects of struvite application on crop yield may be realized in the years following application in addition to the year of application (Thiessen Martens et al., 2022).

The research presented in the current manuscript is the first evaluation at the field scale of the implications for snowmelt runoff P loss following application of struvite and the first evaluation of changes in P losses with snowmelt runoff from organic perennial crops with changes in soil P fertility over time. This research was designed to test the hypotheses that (1) drawdown of soil P over time under an unfertilized perennial forage production system will result in a reduction in potential for losses of P with snowmelt runoff, and (2) as a low‐solubility source of P, struvite can be applied to maintain soil P without increasing losses of P in snowmelt runoff in the years following application.

## 2 MATERIALS AND METHODS

### 2.1 Study site

The present study provides an evaluation of changes in P runoff losses over a 7‐year period (2017–2023) from three field‐scale small watersheds following the conversion of an annual small grains field under organic management to perennial alfalfa (*Medicago sativa* L.)–meadow brome grass (*Bromus riparius* Rehm.) forage (Figure S1). One watershed received biennial addition of struvite to maintain soil fertility, while two others had no P amendment resulting in drawdown of soil P. To further evaluate potential changes in snowmelt runoff P losses following struvite application over a wider range of site conditions, single site‐year applications and monitoring of subsequent snowmelt losses were completed in 2 separate field‐scale watersheds (Figure S2) following conventional annual crops of soybean [*Glycine max* (L.) Merr.] and spring wheat (*Triticum aestivum* L.).

Response over time following struvite application or drawdown was evaluated in a 10.5 ha certified organic field located near Kenton, MB (Figure S1; 50°01′16″, 100°34′02″). Soil in the field is classified under the Canadian system as an Orthic Black Chernozem of the Newdale Series, with a Clay Loam texture (Canadian Agricultural Services Coordinating Committee—Soil Classification Working Group, 1998), and as a Haplocryolls under the USDA soil classification (Soil Survey Staff, 2006). Soils of the Newdale Series are moderately to well drained and formed on calcareous loamy morainal till. Over the last 20 years this field has been maintained under a crop rotation of 3–5 years of alfalfa–grass hay followed by a 3–4 year sequence of annual crops, with a flexible rotation principally of spring wheat (*Triticum aestivum* L.), oats (*Avena sativa* L.), and flax (*Linum usitatissum* L.). The cooperating farm for this research is a mixed beef cattle and grain farm, so manure has been applied to the field every 8–10 years to alleviate soil P deficiencies that have been observed to reduce yields.

The regional climate is humid continental (Dfb, Köppen classification; Kottek et al., 2006), which historically does not exhibit a distinct dry season and has warm summers. The 30‐year precipitation normal is 497.5 mm for annual precipitation and 320 mm for the growing season (May 1–September 30) as reported at the nearest Environment and Climate Change Canada Station with historical weather data (Oakner, MB; 50°06′00″, 100°30′00″). Soils in the region typically reach temperatures below 0°C starting at surface depths (0–15 cm) in November of each year with freezing becoming deeper over time. Thawing of soils over the course of snowmelt tends to occur from the top down, with frozen soils remaining deeper in the soil profile until after snowmelt in March or April. The majority of annual P loss to surface water occurs through snowmelt runoff over a layer of frozen soil (H. Wilson et al., 2019).

Since 2015, a 1.50 ha small watershed (Org 4) draining a portion of the field has been instrumented at the watershed outlet to measure P export in both snowmelt and rainfall runoff. In 2017, measurement of P losses with runoff was initiated in two additional small watersheds (Org 5 and Org 6), draining separate portions of the field (1.36 ha and 0.87 ha). Measurements in these three small watersheds (Figure S3) were first initiated to represent sites having low soil test P as part of a broader regional study to understand drivers of P loss with snowmelt runoff (H. Wilson et al., 2019).

**Core Ideas**

- Drawdown of surface soil P with repeated harvest of perennial hay reduces potential for snowmelt runoff losses.
- Struvite added to soils significantly increased 0–5 cm Olsen P (147 ± 85%).
- Risk of P loss with snowmelt runoff relates more strongly to background Olsen P than to the level after struvite amendment.
- Further research is needed to determine predictors of change in solubility of added struvite P over time.
- At the same level of Olsen P, risk of P loss with snowmelt is lower than predicted if soils contain struvite.

Crop rotation and all farm operations throughout the study field were kept consistent over the entire field area up until the first application of struvite to the treatment watershed (Org 4) in May of 2019. Struvite was applied as granular fertilizer (3‐mm diameter Crystal Green, 5‐28‐0‐10 Mg guaranteed nutrient analysis supplied by Ostara Nutrient Recovery Technologies Inc.) into a newly established stand of alfalfa–grass hay by the cooperating farm using a John Deere 1590 no‐till drill with a target depth of 2.5 cm and a rate of 40 kg P ha^−1^. This target rate was selected to match those previously measured with manure applications utilized on the farm to address soil P deficiencies. Struvite was applied at a rate of 31 kg P ha^−1^ in May 2019 and at a rate of 45 kg P ha^−1^ in October 2020 (Supporting Information Methods). Aside from the two struvite applications, all field operations were kept consistent over the entire study field, resulting in a drawdown of P in the Org 5 and Org 6 watersheds. However, after struvite application, the spatial pattern of operations was altered to ensure the treatment area was harvested separately from the rest of the field to calculate differences in yield and because struvite derived from human wastewater is not currently permitted under the Canadian Organic Standard. As a result, the treatment area had to be removed from organic certification for the duration of the study, with harvested hay from that portion of the field being sold into the conventional market rather than used on farm. At the end of the growing season in 2022, tillage (disc and cultivator) was used to terminate the alfalfa–grass stand, also referred to as breaking.

To evaluate the impact of struvite application on P runoff over a wider range of conditions, one‐time applications of struvite were also completed in two separate field‐scale small watersheds (Con Till 1 and Con Till 4) that are independent of the organic study field and that drain fields where annual grains were grown with conventional production methods (Figure S2). In the years previous to struvite application, MAP was applied annually in each conventional field at the time of seeding. Soils in both fields were Orthic Black Chernozems (or Haplocryolls) with Clay Loam texture and receive tillage periodically for crop residue management. Soil types, management histories, and annual changes in soil P are described in greater detail in both H. Wilson et al. (2025) and H. Wilson et al. (2019) for Con Till 1 and Con Till 4 sites. At the Con Till 1 field site, granular struvite (3 mm) was broadcast at a rate of 36 kg P ha^−1^ and then incorporated using a field cultivator prior to seeding of a soybean crop in 2019. Impact on runoff water chemistry was measured in the spring of 2020. At the Con Till 4 field site granular struvite was broadcast at a rate of 21 kg P ha^−1^ and incorporated by cultivation prior to seeding of a wheat crop in 2021, with impact on runoff water chemistry measured in the spring of 2022. At the Con Till 4 site in 2021, P was also applied as MAP with subsurface application at the time of seeding (4.8 kg P ha^−1^).

### 2.2 Baseline soil characteristics and measurement of soil P

Within each small watershed included in the study, duplicate soil samples were collected annually at three to six locations (6–12 sampling points total). Locations were previously selected as benchmark sites using a stratified design to ensure placement within each of the generalized landforms present (upper, middle, and lower slope and surface depressions) as described in H. F. Wilson et al. (2016) (Figure S1). Baseline soil characterization of the primary study site was completed in 2016 prior to initiating runoff measurements in the three watersheds indicating conditions typical of Newdale Series soils, with soils in the top 15 cm being neutral or slightly alkaline (pH 7.5 ± 0.73) and having relatively high organic carbon content (3.0 ± 0.69%). Soils in the two small watersheds where one‐time struvite applications occurred were classified as a Ramada Clay Loam (Con Till 1) and Dezwood Clay Loam (Con Till 4) with 0–15 cm soil pHs of 6.8 ± 0.32 and 6.2 ± 0.63 as measured prior to struvite application. Further details of watershed delineation and earlier soil characterizations are also available in H. Wilson et al. (2019) where these watersheds were coded as Org 4, Org 5, Org 6, Con Till 1, and Con Till 4.

At each sampling point, soil samples were collected for both 0‐ to 5‐cm and 0‐ to 15‐cm depths. Soil sampling was timed each year to coincide with the end of growing season, after all field operations had been completed by the cooperating farmers and soil temperatures had dropped below 4°C. Soil P was measured using the same methods described in H. F. Wilson et al. (2016). With soil of the study fields being calcareous, the standard regional soil test method was utilized (NaHCO_3_ extractable; Olsen P). Samples were air‐dried, sieved (2 mm), and then ground prior to analysis. Previous research involving these sites has shown a strong correlation of 0–5 cm Olsen P with both water‐extractable soil P and concentrations of P measured in runoff (H. Wilson et al., 2019; H. F. Wilson et al., 2016).

### 2.3 P mass balance and crop yields

For each year of measurement at the organic study site, the balances of P inputs and removals for control and treatment portions of the study field were calculated (Supporting Information Methods). Rates of removal were calculated based on the harvested crop and measured in losses with runoff. A cumulative P mass balance was calculated over the course of the study period for each treatment area by adding the annual mass balance for each year (positive or negative) to a running total. Input by atmospheric deposition was assumed to be small in comparison to other fluxes and consistent across sites. Losses of P through leaching to groundwater were also assumed to be negligible based on very low volume or zero water being collected annually in lysimeters at the Org 4 and Con Till 1 study sites (unpublished data). However, movement of P downward in the soil profile, particularly from the 0‐ to 5‐cm to 5‐ to 15‐cm depths may account for some variability in observed relationships between mass balance and Olsen P.

### 2.4 Measurement of P runoff

Runoff was measured using sharp‐crested v‐notch weirs at the outlet of each of the small watersheds monitored. Water level behind each weir was measured using multiple strategies (pressure transducer, ultrasonic sensor, and photograph time series) to minimize any gaps in the dataset resulting from instrument damage or interference with diurnal freeze and thaw of the runoff water (Figure S3). In the 2017–2023 period at the main study site, surface runoff events occurred annually with spring snowmelt and no instances of rainfall‐driven runoff were observed. Snowmelt runoff was only measured in the year following struvite application at the Con Till 1 site in 2020. At the Con Till 4 site, snowmelt runoff was measured in the spring of 2022 following struvite application in the previous year. Additional detail is provided in H. Wilson et al. (2019) on instruments used in measurement, calculation of flow, and correction of time series data for the effects of ice formation.

Automated water samplers (Sigma 900) were installed at each weir and were programmed to collect samples at a 6‐h time interval prior to the onset of peak flow, at a 4‐h time interval near peak flow, and at a 6‐h interval as flow receded. Sites were visited twice daily during snowmelt runoff: in the early morning (daily onset of flow) and late afternoon (near peak flow). Site conditions were checked with each visit, any ice formed overnight was removed in the morning, and manual samples were collected if automated sampler intake lines had frozen overnight. Samples were collected daily, kept cold, and filtered within 48 h of collection through pre‐combusted glass fiber filters (nominal pore size 0.7 µm). As described in H. Wilson et al. (2019), a 0.7‐µm pore size was utilized after comparison against 0.45 µm duplicates from a variety of runoff samples collected in Manitoba showed no significant difference for either dissolved reactive P (DRP) or total dissolved P (TDP). Filtered samples were kept frozen until analysis for TDP and DRP. An unfiltered sample was also kept frozen until analysis for total P (TP). Prior to colorimetric analysis, sulfuric acid/persulfate digestions were completed for TP and TDP subsamples. Colorimetric analysis for all P forms was completed using the ascorbic acid method. Accuracy of analysis was verified against stated range on the certificate of analysis of external standards included with each analytical run and precision of 5% or less was maintained for each sample.

Prior to calculating loads of each P form, concentration for each 15‐min timestep was calculated by linear interpolation. Load (mass of P lost with runoff per unit area) for each timestep was then calculated as the product of concentration and discharge, with sum of loads for each timestep being the annual total. Since rainfall runoff did not occur over the course of the study, annual total and total for snowmelt were the same. For each year, flow‐weighted mean concentrations (FWMCs) were calculated as load divided by the total volume of runoff measured.

### 2.5 Statistical methods

Interannual differences in Olsen P as measured at the end of each growing season are described using simple statistics. Linear regression was used to evaluate whether a relationship was observed between P mass balance in each watershed as calculated at the end of each growing season and measured Olsen P. Differences in hay yield between control and treatment portions of the study field were evaluated through comparison of measured values and associated error as presented in the Supporting Information Materials. An estimate of error for yield values was generated by multiplying the number of bales harvested by the upper and lower 95th percentiles of the mean mass of bales sampled in a given year. Although this study was not designed to systematically identify the impact of struvite fertilization on yield, the potential importance of environmental conditions (Olsen P, soil moisture, accumulated precipitation, and days of growth) on hay yield was evaluated based on strength of univariate correlation.

The research presented here was primarily designed to identify any changes in potential for P losses with snowmelt runoff from a perennial forage crop where soil P was either allowed to decline over time or where struvite was applied to build and maintain soil P. Companion research over a wide range of cropping systems in Manitoba (82 site‐years) has identified soil P (0–5 cm Olsen P), water yield (volume of runoff per unit area), and soil surface area covered by crop residue as the primary environmental drivers of interannual variability in P concentration and load in snowmelt runoff (H. Wilson et al., 2025). Due to natural variability in weather conditions and changing Olsen P through time, these environmental factors varied widely between years in our study watersheds (Supporting Information Data). For this reason, a simple before‐and‐after paired watershed approach could not be used to define changes in a treatment as compared to a control watershed.

As an alternative to a traditional paired watershed analysis, snowmelt P losses from study watersheds following struvite applications are compared to predictions of load and FWMC using regional models presented in H. Wilson et al. (2025). Site‐years in the current study without application of struvite (P drawdown) were part of the overall H. Wilson et al. (2025) dataset (noted in Supporting Information Data) and exhibited the same drivers of P loss as other annual cropland or perennial forage site‐years regionally. Struvite applications were observed to increase Olsen P, but did not impact water yield or surface area covered by crop residue. To evaluate whether a significant impact of struvite application was observed, measured loads and FWMC after application were compared against predicted values calculated using background 0–5 cm Olsen P measured in the year prior to any application of struvite. Since residual struvite and reaction products in soil have been observed to be detectable using the Olsen P method on dried and ground soils (Gu et al., 2021), predicted loads and FWMC based on measured 0–5 cm Olsen P in years following application were also calculated for comparison to predictions based on Olsen P prior to amendment. Statistical significance of observed differences was defined in comparison to the 95% prediction intervals of the H. Wilson et al. (2025) multiple regression models for FWMCs and load as calculated with JMP 17 statistical software (SAS Institute). Goodness of fit for measured FWMCs and loads following struvite application was also quantified in comparison to predicted values using root mean square error (RMSE). As observed in H. Wilson et al. (2025), water yields and concentrations of P in soil and water do not follow normal distributions. To meet assumptions of normality and for comparison with the predictive model, water yields, loads, and concentrations were all square root transformed prior to analyses.

Given that loads and FWMC are likely to be overpredicted based on measured Olsen P in struvite‐containing soils but possibly underpredicted in comparison to unamended background soil P concentrations, an additional parameter to indicate potential for residual struvite in soil to contribute to snowmelt losses was calculated. For this analysis, the difference between measured 0–5 cm Olsen P after struvite application and the background measured prior to application was used as an indicator of the magnitude of change in Olsen P associated with struvite addition (Δ 0–5 cm Olsen P). To estimate the difference in runoff loss risk (LR) where Olsen P was increased by struvite application as compared to sites where Olsen P was not added, a new parameter (LR) was solved for using the nonlinear fit platform in JMP 17, where all parameters in regional best fit models were kept constant, but for 6 site‐years where residual struvite may have been present in the soil prior to snowmelt, 0–5 cm Olsen P was represented in the model as background 0–5 cm Olsen P + LR (Δ 0–5 cm Olsen P).

## 3 RESULTS

### 3.1 Olsen P as influenced by mass balance

**FIGURE 1:**
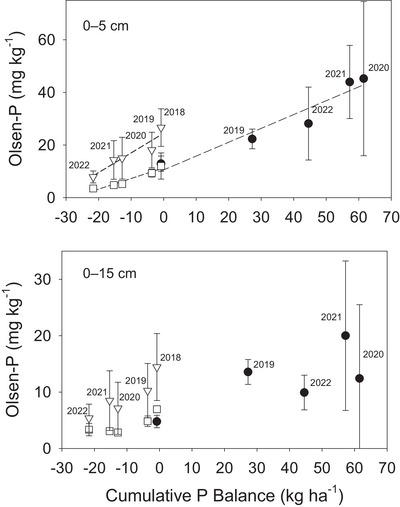
Mean Olsen P and cumulative P balance changes over time for two watersheds without any P amendment (Org 5—Drawdown 1; triangles, Org 6—Drawdown 2, open squares) or with addition of struvite (Org 4—Struvite; filled circles). Error bars indicate 95% confidence intervals. Years of measurement are labeled above points and correspond with all points below each label. For the 0‐ to 5‐cm depth, best‐fit regression lines are shown as a dashed line. The best fit parameters for regression lines are *r* ^2^ of 0.89, 0.88, and 0.92 for Drawdown 1, Drawdown 2, and Struvite watersheds, with *p* < 0.001.

In the organically managed drawdown watersheds, Olsen P in both the 0‐ to 5‐cm and 0‐ to 15‐cm depths of soils declined over time (Table 1; Figure 1). In the struvite watershed, applications in the spring of 2019 and late autumn of 2020 resulted in increased Olsen P concentrations with highest levels observed in the 2020 and 2021 growing seasons. Seasonal availability of water was the primary influence on hay yield over time, with the drawdown and struvite treatments exhibiting similar hay yields at the start of the experiment; however, beginning in 2021, the struvite treatment watershed yielded significantly higher than the drawdown treatment (Supporting Information Material).

**TABLE 1:** P inputs, harvest removal, runoff losses (total P measured in the spring prior to growing season), and overall P balance for an organic alfalfa–grass hay field in Manitoba with either no P inputs or where struvite was applied as a P amendment. The harvested crop from 2019 to 2022 was alfalfa–grass hay, while in 2018, the harvested crop was oat–pea silage, which was seeded as a nurse crop to establish the alfalfa–grass hay.

Year	Fertilizer management	P inputs (kg ha^−1^)		P removal with harvest (kg ha^−1^)^a^		Runoff P losses (kg ha^−1^)^b^		Annual P balance (kg ha^−1^)		Cumulative P balance (kg ha^−1^)	
		Drawdown	Struvite	Drawdown	Struvite	Drawdown	Struvite	Drawdown	Struvite	Drawdown	Struvite
2018	–	0	0	0.80	0.80	0.001, 0.001	0.012	−0.80	−0.812	−0.80	−0.814
2019	Struvite applied to treatment	0	31.0	2.68	2.80	0.088, 0.048	0.097	−2.75	28.1	−3.55	27.2
2020	Struvite applied to treatment	0	45.2	8.86	10.17	0.261, 0.209	0.526	−9.10	34.3	−12.6	61.5
2021	–	0	0	2.57	4.28	0.000, 0.000	0.002	−2.57	−4.3	−15.2	57.3
2022	–	0	0	6.32	12.45	0.148, 0.020	0.169	−6.4	−12.6	−21.6	44.7

^a^ Crop yield and measured P content are detailed in Table S1 and the manuscript text.

^b^ Mean of runoff P losses for the two Drawdown treatment watersheds was used to calculate mass balance. Runoff P loads specific to each watershed are discussed in detail in other parts of the manuscript.

Interannual Olsen P trends were linearly related to changes in the overall mass balance of P addition and removal (Figure 1), particularly at the 0‐ to 5‐cm depth. For the 0‐ to 15‐cm depth, measured Olsen P was significantly more variable in those years with greatest increases in Olsen P following struvite application. This was likely a result of uneven distribution of residual struvite in the soil both laterally and vertically, with 0‐ to 5‐cm samples being of a larger volume and more likely to contain struvite following collection of a slab of soil with a shovel, while 0‐ to 15‐cm soil cores may have hit or missed locations with higher concentration of struvite remaining in soil following initial application in bands. Tillage to terminate the alfalfa–grass stand at the end‐of‐season in 2022 likely resulted in mixing between soil depths.

### 3.2 P loads and concentrations in runoff

Hydrologic conditions varied significantly over the period of observation for this study (2017–2023). At the organic study watersheds, observed water yields ranged from 0 to 95 mm; accumulated snow water equivalent at the time of melt ranged from 20 to 140 mm. At the Con Till 1 study watershed, water yield measured in 2020 was 23 mm. For the Con Till 4 site, water yield measured in 2022 was 153 mm following late‐season snowfall and significant snowpack accumulation.

As previously observed in the region, the majority of P exported during snowmelt was in a dissolved form, with a relatively consistent and small amount of TDP measured in an unreactive form (often assumed to be organic) and of TP measured in a particulate form. DRP was not measured in all site‐years used to develop the model presented in H. Wilson et al. (2025), so comparison of the impact of struvite applications was not possible. However, for site‐years measured at the organically managed field site through time, the linear relationship between FWMC DRP and TDP was linear and with an intercept of zero, this relationship had a slope of 0.86 (*r* ^2 ^= 0.99, *p* < 0.001, *n* = 18). The relationship between TDP and TP was also linear and where intercept was zero, the slope of that relationship was 0.94 (*r* ^2 ^= 0.97, *p* < 0.001, *n* = 18).

### 3.3 Influence of environmental conditions and P management strategies on potential for P runoff losses

**FIGURE 2:**
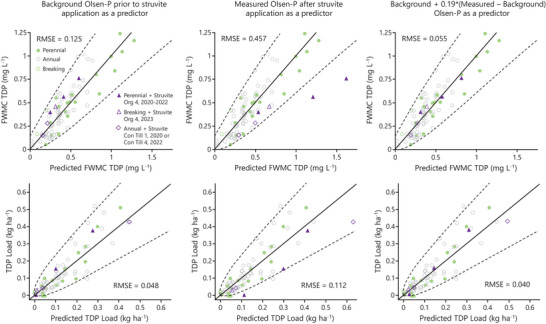
Flow‐weighted mean concentration (FWMC) and load of total dissolved P (FWMC TDP) are plotted against predicted values generated using the regional models for each metric as presented in H. Wilson et al. (2025). Markers shown for site‐years without struvite application indicate data previously presented in H. Wilson et al. (2025) and used in developing each regional model. Solid lines indicate a 1:1 relationship between predicted and measured values. Dashed lines show 95% prediction intervals for each model. Each model included 0–5 cm Olsen P, water yield, and surface area covered by crop residue. Different measures of Olsen P were applied for those site‐years following struvite application as noted in panel titles. Root mean square error indicates the goodness of fit when comparing the 6 site‐years following struvite application to model predictions.

As described in H. Wilson et al. (2025), near‐surface (0–5 cm) Olsen P and water yield were identified as best predictors of both FWMCs and loads of TP and TDP, with surface area covered by crop residues predicting additional variation. Where background Olsen P prior to struvite application is used as a predictor in the regional models for TDP FWMC and load, measured values do not significantly differ from modeled values (within both the 95% and 75% prediction intervals; Table 2; Figure 2). Although differences between measured and modeled values based on background Olsen P were not significantly different from TDP FWMCs or loads, measured values were consistently higher than predicted (Table 2). Where Olsen P measured following struvite application was used as a predictor of potential for runoff losses, measured FWMC and loads were consistently lower than predicted, with FWMC values for 2 of 6 site‐years of data falling below the lower 95% prediction intervals (Table 2; Figure 2). When comparing measured FWMCs and load to predicted values for the 6 site‐years following struvite application, goodness of fit was consistently better (lower RMSE) when using background Olsen P as a predictor as opposed to Olsen P measured for soils containing struvite. Solving for a parameter to estimate runoff LR of Olsen P increased by struvite application (Δ 0–5 cm Olsen P) as compared to Olsen P at sites without struvite, identified a best fit of 0.19 ± 0.09 for TDP FWMC and 0.19 ± 0.19 for load.

**TABLE 2:** Comparison of measured values following application of struvite to those predicted using the regional models developed in H. Wilson et al. (2025) for snowmelt flow weighted mean concentration (FWMC) and load of total dissolved phosphorus (TDP) and total phosphorus (TP). Predictors in each model are 0–5 cm Olsen P, water yield, and surface area covered by crop residue. Values are shown where either background Olsen P measured in the year prior to struvite application or Olsen P measured in any given year and following struvite application were used in each model. Upper or lower 75th and 95th prediction intervals are indicated depending on direction of expected exceedance.

			Values modeled using background 0–5 cm Olsen P			Values modeled using annually measured 0–5 cm Olsen P		
Site	Year	Measured	Predicted	Upper 75th	Upper 95th	Predicted	Lower 75th	Lower 95th
**FWMC TDP (mg L** ^**−1**^ **)**								
Con Till 1	2020	0.149	0.157	0.277	0.384	0.293	**0.169**	0.101
Org 4	2020	0.397	0.248	**0.394**	0.519	0.532	0.360	0.257
Org 4	2021	0.761	0.601	0.830	1.016	1.607	**1.282**	**1.071**
Org 4	2022	0.561	0.413	0.600	0.755	1.201	**0.929**	**0.756**
Org 4	2023	0.456	0.314	0.475	0.611	0.665	**0.472**	0.353
Con Till 4	2022	0.279	0.213	0.354	0.477	0.494	**0.324**	0.224
**Load TDP (kg ha** ^**−1**^ **)**								
Con Till 1	2020	0.042	0.039	0.087	0.133	0.072	0.029	0.010
Org 4	2020	0.376	0.277	0.389	0.480	0.412	0.296	0.225
Org 4	2021	0.002	0.008	0.036	0.069	0.112	**0.054**	**0.025**
Org 4	2022	0.154	0.101	0.174	0.239	0.297	**0.199**	0.140
Org 4	2023	0.030	0.012	0.042	0.075	0.056	0.019	0.005
Con Till 4	2022	0.427	0.451	0.595	0.712	0.627	**0.478**	0.384
**FWMC TP (mg L** ^**−1**^ **)**								
Con Till 1	2020	0.217	0.227	0.373	0.501	0.382	**0.233**	0.149
Org 4	2020	0.555	0.324	**0.495**	0.639	0.635	0.439	0.320
Org 4	2021	0.894	0.719	0.964	1.163	1.774	**1.419**	**1.188**
Org 4	2022	0.618	0.510	0.715	0.884	1.345	**1.046**	**0.854**
Org 4	2023	0.525	0.406	0.593	0.748	0.790	**0.570**	0.434
Con Till 4	2022	0.373	0.283	0.447	0.588	0.592	**0.398**	0.282
**Load TP (kg ha** ^**−1**^ **)**								
Con Till 1	2020	0.061	0.058	0.115	0.167	0.098	0.046	0.021
Org 4	2020	0.526	0.351	**0.476**	0.577	0.500	0.372	0.291
Org 4	2021	0.002	0.008	0.036	0.067	0.113	**0.055**	**0.026**
Org 4	2022	0.169	0.125	0.202	0.269	0.335	**0.230**	0.166
Org 4	2023	0.034	0.017	0.051	0.088	0.066	0.026	0.008
Con Till 4	2022	0.571	0.573	0.732	0.859	0.768	**0.602**	0.495

*Note*: Bold text highlights instances where measured values are outside of prediction intervals.

## 4 DISCUSSION

### 4.1 Does the drawdown of soil P over time under an unfertilized perennial forage production system reduce the potential for losses of P with snowmelt runoff?

As was also observed for annual cropland by Liu, Elliott et al. (2019), the cumulative mass balance of P inputs and outputs appears to be the primary factor controlling the accumulation or depletion of mobile P in soils of perennial hay fields in the region. Biomass removal from perennial forage crops harvested as hay is particularly high, directly contributing to P removal over time in this study (Table 1). The potential for decline in soil P under hay production systems without fertilization is a well‐recognized issue for organic producers in the northern Great Plains (Welsh et al., 2009), but when used to draw down excess soil P, a beneficial reduction in losses of P to downstream aquatic systems is likely to occur (H. Wilson et al., 2025).

Changes in the potential for P loss with snowmelt following drawdown of soil P in the present study are similar to those observed for grass riparian buffers in Finland, where Uusi‐Kämppä (2005) also observed a reduction in accumulated soil P and a reduction of loss with snowmelt following repeated harvest removal of plant biomass. In addition, where P losses in snowmelt runoff and TP leachable from plant residues have been quantified for grain cropping or cover crops (mainly annual or biennial), high rates of retention of leachable P by soil are frequently observed (Liu, Macrae, et al., 2019; Lozier et al., 2017).

The results observed in the current study show the potential for perennial hay to draw down soil P. The analysis presented in H. Wilson et al. (2025) shows that potential for snowmelt runoff losses will be reduced where 0–5 cm Olsen P is reduced, with no significant impact of the presence or absence of perennial vegetation. That analysis included non‐struvite site‐years from the current study as well as those from a wider range of soils in Manitoba (Supporting Information Data). Together, these results indicate that where Olsen P is elevated, drawdown by repeated harvest of perennial hay should be an effective strategy to reduce potential for snowmelt runoff losses of P.

### 4.2 Can struvite be applied to perennial forages to increase yield without increasing losses of P in surface runoff?

Under an organic production system, subsurface application of struvite was observed to be an effective tool in maintaining adequate background soil P and to maintain alfalfa–grass hay yield over time. At the same time, repeated harvest without soil amendment resulted in depletion of soil P and yield potential in unfertilized land areas (Supporting Information Material).

Potential for snowmelt P losses following application of struvite was not significantly higher as compared to predicted losses based on background Olsen P prior to struvite amendment. In addition, FWMCs and loads of P in snowmelt were consistently lower than predicted based on Olsen P measured after struvite applications (Table 2; Figure 2). These patterns suggest that susceptibility of P leaching from soils following application of struvite is lower than where Olsen P has been built over time using other forms of P (MAP or beef manure) as in H. Wilson et al. (2025). These patterns match with the expectation of low water solubility of recently applied struvite and the observations of Thiessen Martens et al. (2023) where low rates of transformation were observed following laboratory incubations of struvite and soil under both warm and cold temperature conditions. However, observed FWMCs were consistently higher than values modeled based on background Olsen P, even if these differences were not statistically significant, suggesting P originally applied as struvite likely undergoes some transformation to a more soluble form over time. This pattern is similar to that observed in recent research by Kokulan et al. (2024) following changes in surface runoff and pore water P concentrations with the application of struvite or MAP to cropland plots in Ontario, where lower P concentrations were also observed in runoff from struvite‐treated plots compared to MAP‐treated plots. However, when compared with control plots receiving no added P, those plots receiving struvite showed a small increase in Olsen P and in water‐extractable P over the course of the study.

Adding a parameter to characterize risk of snowmelt runoff P loss where Olsen P was added with struvite consistently improved accuracy of model predictions (Figure 2). For both FWMC and load of TDP, this parameter was around 0.19 (±0.09 and ±0.19), suggesting an 81 ± 19% lower risk of runoff loss where Olsen P was increased by addition of struvite as compared to where Olsen P has been built to a similar level using other more soluble forms of P. However, this value also indicates that there is a portion of added Olsen P following struvite application that may become water soluble over the late autumn, winter, and early spring months. Since the parameter to estimate LR of added Olsen P by struvite only applied to 6 site‐years, each with different duration of struvite contact with soils, the standard error of LR estimates was relatively high. Further research is needed to improve predictions and to identify drivers of susceptibility of soil P to loss in runoff following struvite application.

The observed relationship through time between the mass balance of P and measured Olsen P (Figure 1) for the struvite treatment watershed suggests that matching annual rates of application to rates of crop removal may be an effective approach to maintaining a target level of background soil P. In addition, potential for runoff P losses and FWMCs was still predictable as a function of soil P where struvite was applied, so principles of good P management can be utilized to prevent losses. Regardless of whether struvite was applied to soils or not, measured snowmelt P losses were higher where 0–5 cm Olsen P was higher. As with other forms of mineral fertilizer used in conventional production or with manure, ensuring that P is applied at an annual rate in balance with anticipated crop demand appears to be of particular importance in preventing accumulation of near‐surface mobile soil P. Application at lower rates can be particularly challenging with manure, due to transport and application costs (Schneider et al., 2019; Withers et al., 2020). In this sense, if applied in organic production systems, granular struvite would offer the unique benefit of greater flexibility to reduce application rate to match crop demand annually and flexibility of longer transport distances due to higher P content relative to manure. The optimum timing (seasonal and annual frequency) and placement depth of struvite should be a topic of future research since the refinement of these aspects of management may further reduce the potential for P loss to runoff while also improving crop P use efficiency.

## 5 CONCLUSION

The results observed in the current study suggest that under cold‐climate conditions, as in warmer climates, the planting of perennial hay along with repeated harvest of biomass is an effective strategy to draw down near‐surface soil P and to reduce the potential for P runoff losses. Overall, the trends of P mass balance, soil P, and runoff P losses observed through time where struvite was applied suggest that, as compared to more soluble forms of P fertilizer, the potential for loss to runoff is lower where soil P is increased with struvite. Changes in snowmelt runoff P losses following increases in Olsen P after struvite application were 81 ± 19% lower than predicted based on a regional model, but further research is needed for validation of P LR estimates. A portion of Olsen P increased by struvite application is still present in a form at risk of loss with snowmelt runoff. As with other P sources, the careful matching of application rate to anticipated crop yield should maintain productivity while also preventing accumulation of excess soil P.

## AUTHOR CONTRIBUTIONS

**Henry Wilson**: Conceptualization; data curation; formal analysis; funding acquisition; investigation; methodology; project administration; writing—original draft. **Kokulan Vivekananthan**: Data curation; formal analysis; writing—review and editing. **Merrin Macrae**: Conceptualization; writing—review and editing. **Jane Elliott**: Data curation; writing—review and editing. **Kim Schneider**: Conceptualization; funding acquisition; project administration; writing—review and editing. **Joanne Thiessen Martens**: Conceptualization; writing—review and editing. **Aaron Glenn**: Data curation; investigation; methodology; writing—review and editing.

## CONFLICT OF INTEREST STATEMENT

The authors declare no conflicts of interest.

## Supporting information



Supplemental material includes site characteristics and map, additional detail on methods used to measure components of annual mass balance, description of crop yields and growing season conditions. Supplemental Data underlying all analyses are available as csv spreadsheets.

Supplementary Material
