# Supplementary material for: Multiyear changes in snowmelt phosphorus runoff with soil P drawdown or application of struvite to an organic forage crop
Source: J Environ Qual. 2025 Oct 23;54(6):1985–95. doi: 10.1002/jeq2.70104 (PMC12593307; doi:10.1002/jeq2.70104)
Supplement: Supplementary file 2 — Supplementary Material [file JEQ2-54-1985-s002.docx]

**Supplemental Material**

**Multiyear changes in snowmelt P runoff losses with drawdown of soil P or application of struvite to an organic forage crop**

Henry Wilson^a*^, Kokulan Vivekananthan^b^, Merrin Macrae^c^, Jane Elliott^d^, Kim Schnieder ^b^, Joanne Thiessen Martens^e^, Aaron Glenn^a^

^a^ Agriculture and Agri-Food Canada, Science and Technology Branch, Brandon Research and Development Centre, Brandon, MB, R7A 5Y3

^b^ Department Of Plant Science, Univ. of Guelph, 50 Stone Rd. E., Guelph, ON, Canada, NIG 2W1

^c^ Department of Geography and Environmental Management, Univ. of Waterloo, 200 University Ave W.,Waterloo, ON, Canada, N2L 3G1

^d^ Environment and Climate Change Canada, National Hydrology Research Centre, Saskatoon, SK S7N 3H5, Canada

^e^ Department Of Soil Science, Univ. of Manitoba, 13 Freedman Cres., Winnipeg, MB, Canada, R3T 2N2

Number of Pages: 9

Number of Tables: 1

Number of Figures: 4

Supplemental material includes site characteristics and maps, additional detail on methods used to measure components of annual mass balances, description of crop yields and growing season conditions, and description of hydrologic anomalies. Supplemental Data underlying all analyses are available as csv spreadsheets.

**Supplemental Figure 1** – Layout of measurement points to evaluate the implications for P runoff of drawing down soil P (Org 5 and Org 6; southern and eastern portions of the field) or maintaining soil P using struvite as a soil amendment (Org 4; northwest corner of the field). The field is located in southwestern Manitoba (50° 01’ 17” N, 100° 34’ 03” W ). Shown in this figure are locations of field boundaries (dotted grey lines), watersheds boundaries (black lines), runoff sampling points (grey stars), soil sampling locations (black points), and landform transitions within watersheds (differentiated by dashed black lines and labelled).

**Supplemental Figure 2** – This map was previously published in Wilson et al. (2025) and indicates locations of single-field small watersheds in Manitoba, Canada where snowmelt P runoff was measured from annual cropland and perennial forages. Separate locations are not visible at the scale presented here for fields in close proximity, so the number of watersheds is noted on the map for each cluster where more than one watershed was sampled. Where one of the watersheds in a cluster received an application of struvite during the present study, site name and location are indicated.

**Supplemental Figure 3** – Monitoring equipment and examples of site conditions at the time of snowmelt runoff for each watershed at the organic study site.

Org 4 - Struvite Treatment Watershed

Org 5 - Drawdown Watershed 1

Org 6 - Drawdown Watershed 2


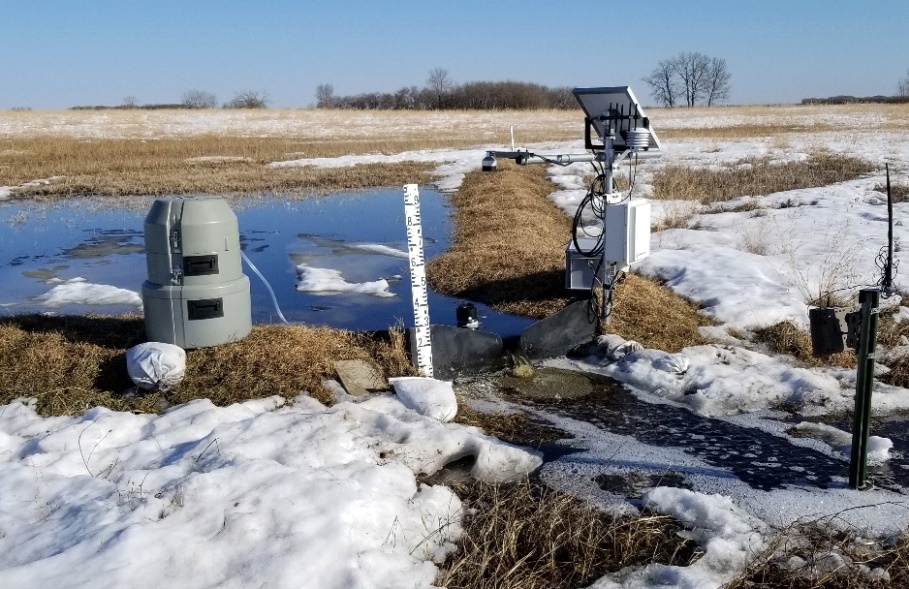

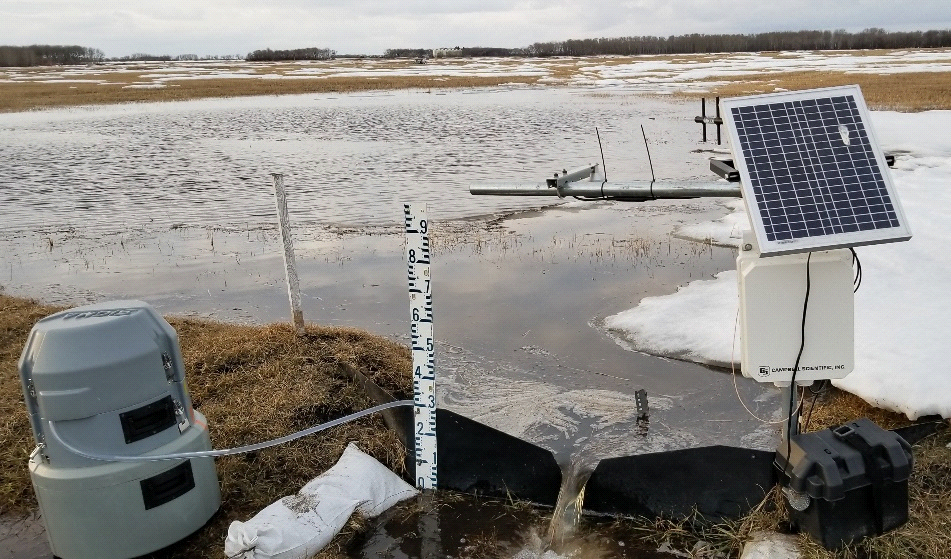

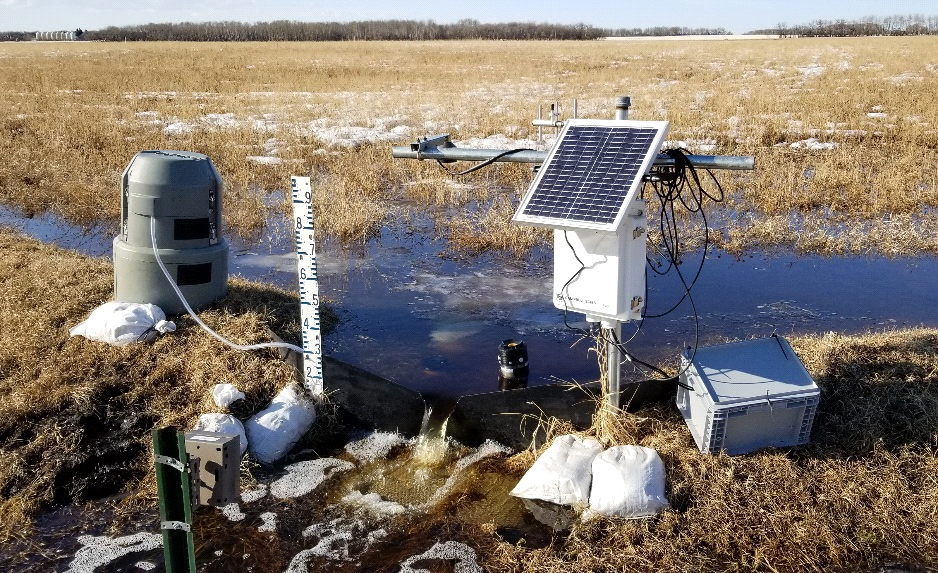


**Supplemental Methods**

**Measurement of struvite application rate**

Actual rate of application was measured based on total mass of product applied (starting mass – any product recovered from the drill after application at a constant rate) divided by area of application (1.8 ha). The area over which struvite was applied and over which resulting crop yields were measured was larger than the treatment watershed area, extending to field boundaries for easier operation of larger equipment (Supplemental Figure 1).

**Crop yields, P content of crops, P application rates, and growing season hydrologic conditions**

Since all portions of the field were managed in the same way until the first application of struvite to the treatment watershed, crop yields up to this date were estimates reported for the field as a whole by the farmer partner. Analysis of crop samples for determination of P content and calculation of P removal with crop harvest was initiated with an Oat/Pea silage crop (under seeded to alfalfa/grass) in 2018, P content was measured for 11 subsamples collected during harvest, with a mean percent P of 0.24±0.05.

After establishment of the alfalfa-grass hay crop, yield and P content of hay was measured within the treatment portion of the field separately from the rest of the field (2019-2022). For both the treatment and control portions of the field, total number of bales produced with each cut of hay was counted and 1-3 bales were set aside by the cooperating producer for analysis of P content. For each portion of the field on each harvest date, these bales were weighed to calculate average mass and then P content in hay was measured from composite of cores collected from the bales. For both silage and hay samples, analysis of P content was completed on dried and ground samples by a commercial laboratory (Agvise Laboratories) by digestion with HNO_3_ and H_2_O_2_ at 150°C and measurement of P using inductively coupled plasma-optical emissions spectroscopy (ICP-OES).

For the first year of measurement in the field (2017), cow-calf beef manure had been applied in the preceding fall (2016) by the cooperating farm at a rate of 31.1 kg ha^-1^ as P, to maintain a minimum level of background fertility. Application rate was determined by collection of manure on 2 m x 2 m plastic sheets placed in 4 locations throughout the field prior to application. Subsamples of manure were kept frozen and sent to a commercial laboratory (A&L Canada Laboratories Inc.) for as received (wet) determination of P content by ICP-OES following nitric-hydrochloric acid digestion. In later years with granular struvite application (2019 and 2020), final P application rate was calculated based on mass of product applied, guaranteed minimum P content as reported by the supplier (Crystal Green^®^, 5-28-0-10Mg), and area of application.

For the growing seasons in which alfalfa-grass was grown at the site (2019-2022), volumetric soil water content over the growing season was also measured at 10cm, 20cm, and 50cm depths at a location with the same soil type, under alfalfa-grass, and within 500 m of the study field (Model CS655 Campbell Scientific, Inc.). Daily precipitation was also measured at this location (Model TR525, Texas Electronics Inc., Dallas, USA) to characterize accumulation over the growing season for comparison to annual normal values.

**Supplemental Results**

**Growing season hydrologic conditions and crop yield**

The intent of this analysis was to identify potential drivers of significant interannual variability in yield that appeared to be largely independent of struvite treatments and to identify hypotheses regarding the influence of environmental conditions on the agronomic impacts of struvite application that might be evaluated in the future through plot scale experiments with a higher degree of replication.

Growing season precipitation (May 1^st^ to September 30^th^) for the 2019 to 2023 period ranged from 71 to 129% of the 30-year normal of 320 mm. Lowest growing season precipitation amounts were recorded in 2023 (71%, 227 mm) and 2021 (81%, 257 mm). In general, observed soil moisture varied seasonally with expected pattern of evapotranspiration and observed rainfall, with greatest variability through time observed at shallower depths (Supplemental Table 1).

Yields of alfalfa-grass hay varied widely depending on both the environmental conditions at the time of and leading up to harvest, with highest yields observed in 2022 and lowest yields observed in 2021 (Supplemental Table 1). Significant correlation exists between interrelated measures of hydrologic condition (soil moisture, days of hay growth, and rainfall prior to cut) making the identification of causality in comparison with hay yield beyond the scope of the current paper; however, a clear trend toward higher yields with higher rainfall accumulation prior to harvest is evident (Supplemental Figure 3). Although yields for the two dates on which a second cut of hay was harvested are also presented in Supplemental Figure 3 for comparison, the overall increases in yield with greater rainfall accumulation are obvious only for first cut hay with longer periods of growth and it seems more likely that second cut hay yield may related more closely to the presence or absence of late-season moisture deficit (Supplemental Table 1).

Although the predominant driver of hay yield over the course of this study appears to be the availability of water, soil P availability did appear to have an important secondary influence. In the first years of measurement (2019 and 2020), yields for each treatment area were similar; however, in the last two years of the experiment (2021 and 2022) the area of the field receiving P amendment as struvite significantly outyielded the portion of the field where soil P was allowed to decline (Supplemental Table 1, Figure 3). This shift corresponded to the greatest estimated differences in available soil P between each treatment area and of a decline in soil P in the drawdown portion of the field to levels low enough to limit productivity.

In the fall of 2022 the study field received tillage and was converted back into the annual grain cropping phase of the rotation. In the spring of 2023, wheat was harvested separately for the struvite and drawdown areas of the field with yield being reported by the cooperating producer. Dry growing conditions resulted in wheat yields around 50% of those previously recorded on the farm under more favourable moisture conditions and without recent soil amendment, but were slightly higher in the portion of the field where struvite was previously applied (1.00 t ha^-1^) as compared with the drawdown area (0.74 t ha^-1^).

**Supplemental Table 1**- Yields and environmental conditions as measured in areas of an alfalfa-grass hay field where either no soil P was added to compensate for removal (drawdown) or where struvite was added as a soil P amendment.

| Harvest Date | Crop | | Yield  (kg ha^-1^) † | | Crop P content (%)‡ | | 0-15cm  Olsen-P  (mg kg^-1^) # | | Volumetric water content (%) | | | Days of hay growth § | Rainfall prior to hay cut (mm) § |
| --- | --- | --- | --- | --- | --- | --- | --- | --- | --- | --- | --- | --- | --- |
|  |  | Drawdown | | Struvite | Drawdown | Struvite | Drawdown | Struvite | 10cm | 20cm | 50cm |  |  |
| Jul 7, 2019 | alfalfa – grass | | 1674  ±67.9 | 1556  ±63.1 | 0.16 | 0.18 | 10.7 | 8.0 | 22.6 | 8.7 | 19.9 | 67 | 122 |
| Jul 13, 2020 | alfalfa – grass | | 4536  ±187 | 4044  ±162 | 0.13 | 0.16 | 8.4 | 13.0 | 38.4 | 24.8 | 35.5 | 73 | 202 |
| Aug 15, 2020 | alfalfa – grass | | 1995  ±79.8 | 2178  ±86.8 | 0.16 | 0.17 | 7.8 | 12.8 | 25.9 | 16.0 | 25.4 | 33 | 82 |
| Jun 30, 2021 | alfalfa – grass | | 1539 ±46.9 | 2425  ±74.5 | 0.17 | 0.18 | 6.1 | 14.8 | 17.2 | 17.2 | 30.0 | 60 | 128 |
| Jul 12, 2022 | alfalfa – grass | | 4903 ±250 | 7150 ±287 | 0.12 | 0.15 | 5.4 | 16.0 | 28.8 | 19.8 | 34.3 | 72 | 291 |
| Sep 6, 2022 | alfalfa - grass | | 515 ±19.1 | 935  ±34.7 | 0.18 | 0.18 | 5.7 | 12.9 | 13.1 | 9.0 | 23.1 | 56 | 89 |

†Yield of hay represents total biomass harvested. 95% confidence interval for hay yield was calculated by multiplying the 95% confidence interval of mean bale mass by number of bales harvested.

‡Crop P content represents a single value for composite samples collected from multiple bales from each treatment.

§For first cut hay days of hay growth is estimated from first rainfall after snowmelt to harvest date. For second cut hay, days of growth is estimated from date of first cut to harvest date of second cut. Accumulated rainfall is the sum of rainfall received over the same time periods as days of growth.

# Olsen-P was only measured once per year in late autumn. It was assumed that content on May 1^st^ was the same as that measured at the end of the previous growing season and linear interpolation between start and end of the growing season was used to estimate Olsen-P content on each harvest date.

**Supplemental Figure 4** – Yields of alfalfa-grass hay as related to rainfall received over the period of growth prior to harvest. Total yields were calculated based on total number of bales collected and average mass of bales harvested, with error bars estimated based on variation in measured bale weights. Solid black points indicate yields measured in the portion of the field receiving struvite as a soil P amendment in spring of 2019 and then in late autumn of 2020. Open points indicate yield measured in the portion of the study field where no P amendments were added between 2017 and 2023, leading to a drawdown of soil P. Dates of harvest are noted near each set of points.
